# Supplementary material for: Changes in sprint performance and sagittal plane kinematics after heavy resisted sprint training in professional soccer players
Source: PeerJ. 2020 Dec 15;8:e10507. doi: 10.7717/peerj.10507 (PMC7747683; doi:10.7717/peerj.10507)
Supplement: Supplemental Information 2 — TE: Typical error, MDC: Minimal detectable change, CV:Coefficient of variation, ICC: Intraclass correlation coefficient. Hz: Hertz, CM: Center of mass. [file peerj-08-10507-s002.docx]

|  | | | | Touchdown | | | | | Toe-off | | | | |
| --- | --- | --- | --- | --- | --- | --- | --- | --- | --- | --- | --- | --- | --- |
|  | Contact time | Step Hz | Step length | CM distance | CM angle | Trunk angle | Hip angle | Contralateral hip angle | CM distance | CM angle | Trunk angle | Hip angle | Contralateral hip angle |
| TE | 0.00 | 0.01 | 0.01 | 0.01 | 0.50 | 1.03 | 1.94 | 1.87 | 0.01 | 0.23 | 0.81 | 1.86 | 0.86 |
| TE lower | 0.00 | 0.01 | 0.01 | 0.01 | 0.36 | 0.74 | 1.39 | 1.34 | 0.01 | 0.17 | 0.58 | 1.33 | 0.62 |
| TE upper | 0.00 | 0.02 | 0.02 | 0.02 | 0.82 | 1.70 | 3.21 | 3.09 | 0.02 | 0.38 | 1.34 | 3.07 | 1.42 |
| MDC % | 2.59 | 0.70 | 2.84 | -40.96 | 1.48 | 6.13 | 5.17 | 3.31 | 3.63 | 1.37 | 4.92 | 3.05 | 2.83 |
| CV % | 0.28 | 0.09 | 0.57 | -63.37 | 0.37 | 1.60 | 1.42 | 0.91 | 0.76 | 0.39 | 1.30 | 0.88 | 0.87 |
| CV upper | -0.67 | -0.16 | -0.47 | -181.06 | -0.01 | 0.24 | 0.25 | 0.21 | -0.25 | 0.10 | 0.26 | 0.30 | 0.36 |
| CV lower | 0.70 | 0.20 | 1.02 | -11.79 | 0.53 | 2.20 | 1.94 | 1.22 | 1.20 | 0.52 | 1.76 | 1.14 | 1.10 |
| ICC | 0.99 | 1.00 | 0.98 | 0.97 | 0.98 | 0.97 | 0.93 | 0.95 | 0.94 | 0.98 | 0.97 | 0.92 | 0.97 |
| ICC intra lower | 0.96 | 0.99 | 0.95 | 0.90 | 0.92 | 0.89 | 0.79 | 0.85 | 0.81 | 0.92 | 0.90 | 0.77 | 0.89 |
| ICC intra upper | 1.00 | 1.00 | 1.00 | 0.99 | 0.99 | 0.99 | 0.98 | 0.99 | 0.98 | 0.99 | 0.99 | 0.98 | 0.99 |
