# Supplementary material for: Changes in sprint performance and sagittal plane kinematics after heavy resisted sprint training in professional soccer players
Source: PeerJ. 2020 Dec 15;8:e10507. doi: 10.7717/peerj.10507 (PMC7747683; doi:10.7717/peerj.10507)
Supplement: Supplemental Information 3 — TE: Typical error, MDC: Minimal detectable change, CV:Coefficient of variation, ICC: Intraclass correlation coefficient. Hz: Hertz, CM: Center of mass [file peerj-08-10507-s003.docx]

|  | | | | Touchdown | | | | | Toe-off | | | | |
| --- | --- | --- | --- | --- | --- | --- | --- | --- | --- | --- | --- | --- | --- |
|  | Contact time | Step Hz | Step length | CM distance | CM angle | Trunk angle | Hip angle | Contralateral hip angle | CM distance | CM angle | Trunk angle | Hip angle | Contralateral hip angle |
| TE | 0.01 | 0.14 | 0.02 | 0.00 | 1.73 | 2.57 | 4.32 | 4.46 | 0.02 | 0.75 | 2.30 | 2.97 | 2.94 |
| TE lower | 0.01 | 0.10 | 0.02 | 0.00 | 1.24 | 1.84 | 3.10 | 3.20 | 0.01 | 0.54 | 1.65 | 2.13 | 2.11 |
| TE upper | 0.01 | 0.22 | 0.04 | 0.01 | 2.86 | 4.24 | 7.13 | 7.37 | 0.03 | 1.24 | 3.79 | 4.90 | 4.85 |
| MDC % | 12.39 | 8.56 | 5.63 | -29.23 | 5.18 | 14.95 | 11.35 | 7.91 | 5.58 | 4.39 | 13.56 | 4.85 | 9.68 |
| CV % | 3.53 | 2.30 | 1.46 | -54.80 | 1.52 | 4.02 | 3.24 | 2.27 | 1.66 | 1.40 | 3.90 | 1.33 | 2.62 |
| CV lower | 0.78 | 0.45 | 0.05 | -153.48 | 0.09 | 0.62 | 0.96 | 0.46 | 0.56 | 0.61 | 1.11 | 0.23 | 0.66 |
| CV upper | 4.74 | 3.12 | 2.08 | -11.55 | 2.15 | 5.52 | 4.24 | 3.06 | 2.14 | 1.75 | 5.12 | 1.81 | 3.48 |
| ICC | 0.47 | 0.41 | 0.89 | 0.99 | 0.73 | 0.64 | 0.75 | 0.51 | 0.87 | 0.76 | 0.60 | 0.62 | 0.74 |
| ICC intra lower | -0.08 | -0.15 | 0.68 | 0.98 | 0.32 | 0.16 | 0.35 | -0.03 | 0.64 | 0.38 | 0.10 | 0.13 | 0.34 |
| ICC intra upper | 0.80 | 0.77 | 0.97 | 1.00 | 0.91 | 0.87 | 0.91 | 0.82 | 0.96 | 0.92 | 0.86 | 0.87 | 0.91 |
