# Supplementary material for: Changes in sprint performance and sagittal plane kinematics after heavy resisted sprint training in professional soccer players
Source: PeerJ. 2020 Dec 15;8:e10507. doi: 10.7717/peerj.10507 (PMC7747683; doi:10.7717/peerj.10507)
Supplement: Supplemental Information 4 — TE: Typical error, MDC: Minimal detectable change, CV:Coefficient of variation, ICC: Intraclass correlation coefficient. Hz: Hertz, CM: Center of mass [file peerj-08-10507-s004.docx]

|  | | | | Touchdown | | | | | Toe-off | | | | |
| --- | --- | --- | --- | --- | --- | --- | --- | --- | --- | --- | --- | --- | --- |
|  | Contact time | Step Hz | Step length | CM distance | CM angle | Trunk angle | Hip angle | Contralateral hip angle | CM distance | CM angle | Trunk angle | Hip angle | Contralateral hip angle |
| TE | 0.01 | 0.09 | 0.02 | 0.00 | 0.79 | 2.39 | 3.85 | 3.29 | 0.01 | 0.47 | 1.79 | 2.28 | 2.15 |
| TE lower | 0.00 | 0.07 | 0.01 | 0.00 | 0.57 | 1.71 | 2.76 | 2.36 | 0.01 | 0.33 | 1.29 | 1.63 | 1.54 |
| TE upper | 0.01 | 0.15 | 0.03 | 0.01 | 1.30 | 3.94 | 6.35 | 5.43 | 0.02 | 0.77 | 2.96 | 3.76 | 3.56 |
| MDC % | 9.32 | 5.71 | 4.89 | -55.67 | 2.36 | 14.21 | 10.15 | 5.85 | 4.76 | 2.75 | 10.81 | 3.73 | 7.11 |
| CV % | 2.38 | 2.11 | 1.20 | -63.68 | 0.60 | 3.67 | 2.87 | 1.35 | 1.28 | 0.75 | 2.85 | 1.10 | 1.87 |
| CV lower | -0.17 | 1.01 | -0.17 | -172.33 | -0.13 | 0.37 | 0.49 | -0.28 | 0.04 | 0.13 | 0.34 | 0.36 | 0.27 |
| CV upper | 3.71 | 2.68 | 1.91 | -6.77 | 0.95 | 5.40 | 4.12 | 2.20 | 1.92 | 1.08 | 4.17 | 1.49 | 2.71 |
| ICC | 0.74 | 0.88 | 0.94 | 0.97 | 0.95 | 0.81 | 0.87 | 0.78 | 0.91 | 0.92 | 0.83 | 0.88 | 0.82 |
| ICC intra lower | 0.35 | 0.65 | 0.82 | 0.91 | 0.84 | 0.50 | 0.62 | 0.43 | 0.73 | 0.75 | 0.54 | 0.66 | 0.52 |
| ICC intra upper | 0.91 | 0.96 | 0.98 | 0.99 | 0.98 | 0.94 | 0.96 | 0.93 | 0.97 | 0.97 | 0.95 | 0.96 | 0.97 |
