# Supplementary material for: Changes in sprint performance and sagittal plane kinematics after heavy resisted sprint training in professional soccer players
Source: PeerJ. 2020 Dec 15;8:e10507. doi: 10.7717/peerj.10507 (PMC7747683; doi:10.7717/peerj.10507)
Supplement: Supplemental Information 5 — TE: Typical error, MDC: Minimal detectable change, CV:Coefficient of variation, ICC: Intraclass correlation coefficient. Hz: Hertz, CM: Center of mass [file peerj-08-10507-s005.docx]

|  | | | | Touchdown | | | | | Toe-off | | | | |
| --- | --- | --- | --- | --- | --- | --- | --- | --- | --- | --- | --- | --- | --- |
|  | Contact time | Step Hz | Step length | CM distance | CM angle | Trunk angle | Hip angle | Contralateral hip angle | CM distance | CM angle | Trunk angle | Hip angle | Contralateral hip angle |
| TE | 0.00 | 0.06 | 0.01 | 0.01 | 0.57 | 0.50 | 1.26 | 2.94 | 0.01 | 0.53 | 0.94 | 1.18 | 1.92 |
| TE lower | 0.00 | 0.04 | 0.01 | 0.01 | 0.38 | 0.33 | 0.83 | 1.94 | 0.01 | 0.35 | 0.62 | 0.78 | 1.27 |
| TE upper | 0.00 | 0.12 | 0.02 | 0.03 | 1.16 | 1.01 | 2.57 | 5.98 | 0.02 | 1.08 | 1.90 | 2.41 | 3.90 |
| MDC % | 5.12 | 3.61 | 1.76 | -11.62 | 1.43 | 1.82 | 2.60 | 4.76 | 4.84 | 2.59 | 3.42 | 1.64 | 5.02 |
| CV % | 1.58 | 0.83 | 0.49 | -2.25 | 0.46 | 0.52 | 0.55 | 1.17 | 1.43 | 0.73 | 0.88 | 0.37 | 0.93 |
| CV lower | 0.17 | -0.21 | 0.00 | -3.88 | 0.22 | 0.15 | -0.12 | -0.33 | 0.56 | 0.22 | 0.04 | -0.10 | -0.61 |
| CV upper | 2.20 | 1.29 | 0.70 | -1.53 | 0.57 | 0.69 | 0.84 | 1.83 | 1.81 | 0.96 | 1.25 | 0.58 | 1.60 |
| ICC | 0.92 | 0.95 | 0.99 | 0.93 | 0.96 | 0.98 | 0.99 | 0.98 | 0.90 | 0.96 | 0.88 | 0.98 | 0.83 |
| ICC intra lower | 0.67 | 0.77 | 0.97 | 0.68 | 0.81 | 0.91 | 0.93 | 0.89 | 0.58 | 0.83 | 0.51 | 0.88 | 0.38 |
| ICC intra upper | 0.98 | 0.99 | 1.00 | 0.98 | 0.99 | 1.00 | 1.00 | 1.00 | 0.98 | 0.99 | 0.97 | 1.00 | 0.96 |
|  |  | | | | | | | | | | | | |
|  |  |  |  |  |  |  |  |  |  |  |  |  |  |
