# Supplementary material for: Changes in sprint performance and sagittal plane kinematics after heavy resisted sprint training in professional soccer players
Source: PeerJ. 2020 Dec 15;8:e10507. doi: 10.7717/peerj.10507 (PMC7747683; doi:10.7717/peerj.10507)
Supplement: Supplemental Information 6 — TE: Typical error, MDC: Minimal detectable change, CV:Coefficient of variation, ICC: Intraclass correlation coefficient. Hz: Hertz, CM: Center of mass. [file peerj-08-10507-s006.docx]

|  | | | | Touchdown | | | | | Toe-off | | | | |
| --- | --- | --- | --- | --- | --- | --- | --- | --- | --- | --- | --- | --- | --- |
|  | Contact time | Step Hz | Step length | CM distance | CM angle | Trunk angle | Hip angle | Contralateral hip angle | CM distance | CM angle | Trunk angle | Hip angle | Contralateral hip angle |
| TE | 0.01 | 0.11 | 0.04 | 0.01 | 0.96 | 1.11 | 2.97 | 3.27 | 0.01 | 0.76 | 1.13 | 1.24 | 1.89 |
| TE lower | 0.00 | 0.07 | 0.03 | 0.01 | 0.65 | 0.75 | 2.00 | 2.21 | 0.01 | 0.52 | 0.76 | 0.84 | 1.28 |
| TE upper | 0.01 | 0.21 | 0.07 | 0.03 | 1.84 | 2.12 | 5.69 | 6.26 | 0.03 | 1.46 | 2.17 | 2.38 | 3.63 |
| MDC % | 12.60 | 6.68 | 5.87 | -10.97 | 2.38 | 3.96 | 6.14 | 5.38 | 6.60 | 3.65 | 3.99 | 1.72 | 4.89 |
| CV % | 3.18 | 1.35 | 1.52 | -2.85 | 0.60 | 1.09 | 1.42 | 1.64 | 1.81 | 0.92 | 1.11 | 0.51 | 0.98 |
| CV lower | 0.07 | -0.42 | -0.22 | -5.27 | 0.03 | 0.28 | -0.18 | 0.52 | 0.18 | 0.04 | 0.22 | 0.19 | -0.50 |
| CV upper | 4.54 | 2.13 | 2.28 | -1.79 | 0.85 | 1.45 | 2.12 | 2.13 | 2.52 | 1.30 | 1.49 | 0.65 | 1.63 |
| ICC | 0.34 | 0.92 | 0.92 | 0.91 | 0.89 | 0.98 | 0.85 | 0.96 | 0.85 | 0.90 | 0.92 | 0.94 | 0.94 |
| ICC intra lower | -0.37 | 0.70 | 0.69 | 0.64 | 0.60 | 0.90 | 0.47 | 0.84 | 0.48 | 0.63 | 0.68 | 0.75 | 0.75 |
| ICC intra upper | 0.80 | 0.98 | 0.98 | 0.98 | 0.97 | 0.99 | 0.96 | 0.99 | 0.97 | 0.98 | 0.98 | 0.99 | 0.99 |
