# Supplementary material for: Changes in sprint performance and sagittal plane kinematics after heavy resisted sprint training in professional soccer players
Source: PeerJ. 2020 Dec 15;8:e10507. doi: 10.7717/peerj.10507 (PMC7747683; doi:10.7717/peerj.10507)
Supplement: Supplemental Information 7 — TE: Typical error, MDC: Minimal detectable change, CV:Coefficient of variation, ICC: Intraclass correlation coefficient. Hz: Hertz, CM: Center of mass. [file peerj-08-10507-s007.docx]

|  | | | | Touchdown | | | | | Toe-off | | | | |
| --- | --- | --- | --- | --- | --- | --- | --- | --- | --- | --- | --- | --- | --- |
|  | Contact time | Step Hz | Step length | CM distance | CM angle | Trunk angle | Hip angle | Contralateral hip angle | CM distance | CM angle | Trunk angle | Hip angle | Contralateral hip angle |
| TE | 0.00 | 0.11 | 0.03 | 0.02 | 1.06 | 0.86 | 1.95 | 2.22 | 0.02 | 0.80 | 0.77 | 1.28 | 1.41 |
| TE lower | 0.00 | 0.07 | 0.02 | 0.01 | 0.72 | 0.58 | 1.32 | 1.50 | 0.01 | 0.54 | 0.52 | 0.87 | 0.95 |
| TE upper | 0.01 | 0.21 | 0.06 | 0.03 | 2.03 | 1.66 | 3.73 | 4.26 | 0.03 | 1.53 | 1.48 | 2.46 | 2.71 |
| MDC % | 10.89 | 6.60 | 4.53 | -12.06 | 2.64 | 3.15 | 4.03 | 3.60 | 8.27 | 3.87 | 2.79 | 1.77 | 3.67 |
| CV % | 2.49 | 1.54 | 0.97 | -3.00 | 0.64 | 0.75 | 0.83 | 1.12 | 2.27 | 1.05 | 0.78 | 0.45 | 0.76 |
| CV lower | -0.85 | -0.32 | -0.74 | -6.10 | -0.03 | -0.05 | -0.47 | 0.40 | -0.01 | 0.11 | 0.07 | 0.03 | -0.40 |
| CV upper | 4.24 | 2.51 | 1.87 | -1.38 | 1.00 | 1.16 | 1.52 | 1.50 | 3.47 | 1.54 | 1.15 | 0.67 | 1.37 |
| ICC | 0.50 | 0.89 | 0.96 | 0.84 | 0.89 | 0.97 | 0.94 | 0.99 | 0.84 | 0.91 | 0.96 | 0.95 | 0.94 |
| ICC intra lower | -0.20 | 0.59 | 0.84 | 0.44 | 0.58 | 0.87 | 0.76 | 0.94 | 0.45 | 0.65 | 0.84 | 0.79 | 0.77 |
| ICC intra upper | 0.86 | 0.97 | 0.99 | 0.96 | 0.97 | 0.99 | 0.99 | 1.00 | 0.96 | 0.98 | 0.99 | 0.99 | 0.99 |
