# Supplementary material for: Changes in sprint performance and sagittal plane kinematics after heavy resisted sprint training in professional soccer players
Source: PeerJ. 2020 Dec 15;8:e10507. doi: 10.7717/peerj.10507 (PMC7747683; doi:10.7717/peerj.10507)
Supplement: Supplemental Information 8 — TE: Typical error, MDC: Minimal detectable change, CV:Coefficient of variation, ICC: Intraclass correlation coefficient. W: Watt, N = Newtons, kg; kilogram, m: meter, s; second, FV: Force-Velocity. [file peerj-08-10507-s008.docx]

|  | | |  |  |  |  |  |  |  |  |  |
| --- | --- | --- | --- | --- | --- | --- | --- | --- | --- | --- | --- |
|  | Vmax theoretical V0 (m/s) | Fmax theoretical F0 (N/kg) | Max ratio of forces (%) | Mean ratio of forces on 10 m (%) | Max Horizontal Power Pmax (W/kg) | Time @ 5 m (s) | Time @ 10 m (s) | Time @ 20 m (s) | Time @ 30 m (s) | Top speed (m/s) | FV-slope with N/kg instead of N |
| TE | 0.09 | 0.27 | 0.51 | 1.48 | 0.51 | 0.02 | 0.02 | 0.03 | 0.03 | 0.06 | 0.04 |
| TE lower | 0.06 | 0.20 | 0.36 | 1.06 | 0.36 | 0.01 | 0.02 | 0.02 | 0.02 | 0.04 | 0.03 |
| TE upper | 0.15 | 0.45 | 0.84 | 2.44 | 0.84 | 0.03 | 0.04 | 0.05 | 0.05 | 0.10 | 0.06 |
| MDC | 0.25 | 0.76 | 1.41 | 4.10 | 1.40 | 0.05 | 0.07 | 0.08 | 0.08 | 0.17 | 0.10 |
| MDC % | 2.72 | 10.19 |  |  | 8.32 | 3.86 | 3.22 | 2.33 | 1.68 | 1.94 | -12.40 |
| CV % | 0.75 | 3.12 | 1.12 | 1.72 | 2.55 | 1.13 | 1.01 | 0.70 | 0.51 | 0.51 | -3.80 |
| CV lower | 0.19 | 1.41 | 0.16 | 0.57 | 1.16 | 0.39 | 0.51 | 0.26 | 0.21 | 0.07 | -5.90 |
| CV upper | 1.00 | 3.87 | 1.76 | 3.05 | 3.16 | 1.45 | 1.22 | 0.90 | 0.64 | 0.70 | -2.88 |
| ICC | 0.97 | 0.83 | 0.94 | 0.80 | 0.94 | 0.86 | 0.90 | 0.95 | 0.97 | 0.98 | 0.66 |
| ICC intra lower | 0.89 | 0.54 | 0.82 | 0.46 | 0.82 | 0.61 | 0.70 | 0.86 | 0.92 | 0.93 | 0.21 |
| ICC intra upper | 0.99 | 0.95 | 0.98 | 0.93 | 0.98 | 0.96 | 0.97 | 0.99 | 0.99 | 0.99 | 0.88 |
