# Supplementary material for: Changes in sprint performance and sagittal plane kinematics after heavy resisted sprint training in professional soccer players
Source: PeerJ. 2020 Dec 15;8:e10507. doi: 10.7717/peerj.10507 (PMC7747683; doi:10.7717/peerj.10507)
Supplement: Supplemental Information 9 — TE: Typical error, MDC: Minimal detectable change, CV:Coefficient of variation, ICC: Intraclass correlation coefficient. W: Watt, N = Newtons, kg; kilogram, m: meter, s; second, FV: Force-Velocity. [file peerj-08-10507-s009.docx]

|  | | |  |  |  |  |  |  |  |  |  |
| --- | --- | --- | --- | --- | --- | --- | --- | --- | --- | --- | --- |
|  | Vmax theoretical V0 (m/s) | Fmax theoretical F0 (N/kg) | Max ratio of forces  (%) | Mean ratio of forces on 10 m (%) | Max Horizontal Power Pmax (W/kg) | Time @ 5 m (s) | Time @ 10 m (s) | Time @ 20 m (s) | Time @ 30 m (s) | Top speed (m/s) | FV-slope with N/kg instead of N |
| TE | 0.10 | 0.24 | 0.59 | 1.80 | 0.40 | 0.02 | 0.02 | 0.02 | 0.03 | 0.06 | 0.02 |
| TE lower | 0.07 | 0.16 | 0.38 | 1.16 | 0.26 | 0.01 | 0.01 | 0.01 | 0.02 | 0.04 | 0.01 |
| TE upper | 0.22 | 0.54 | 1.30 | 3.96 | 0.87 | 0.04 | 0.05 | 0.05 | 0.06 | 0.14 | 0.05 |
| MDC | 0.28 | 0.68 | 1.64 | 4.99 | 1.10 | 0.06 | 0.06 | 0.06 | 0.07 | 0.18 | 0.06 |
| MDC % | 3.13 | 9.53 |  |  | 6.97 | 4.00 | 2.78 | 1.71 | 1.50 | 2.10 | -7.37 |
| CV % | 0.87 | 2.87 | 1.76 | 2.95 | 2.13 | 1.17 | 0.87 | 0.53 | 0.45 | 0.57 | -3.64 |
| CV lower | 0.24 | 0.78 | 0.58 | 0.58 | 0.49 | 0.23 | 0.25 | 0.15 | 0.10 | 0.14 | -6.34 |
| CV upper | 1.15 | 3.79 | 2.27 | 3.98 | 2.85 | 1.58 | 1.14 | 0.70 | 0.61 | 0.77 | -2.46 |
| ICC | 0.92 | 0.60 | 0.75 | 0.23 | 0.87 | 0.62 | 0.80 | 0.94 | 0.92 | 0.97 | 0.49 |
| ICC intra lower | 0.63 | -0.09 | 0.10 | -0.57 | 0.41 | -0.16 | 0.20 | 0.70 | 0.62 | 0.82 | -0.33 |
| ICC intra upper | 0.99 | 0.86 | 0.95 | 0.81 | 0.98 | 0.92 | 0.96 | 0.99 | 0.99 | 0.99 | 0.89 |
