# Supplementary material for: Changes in sprint performance and sagittal plane kinematics after heavy resisted sprint training in professional soccer players
Source: PeerJ. 2020 Dec 15;8:e10507. doi: 10.7717/peerj.10507 (PMC7747683; doi:10.7717/peerj.10507)
Supplement: Supplemental Information 10 — Values: Tec = Technical Training, Tac = Tactical Training, LB = Lower Body, MB = Mixed full body training, CG = Condition Games, Rec = Recovery Training, SP = Spinning, RE = Resisted Sprinting Training, M = Match. [file peerj-08-10507-s010.docx]

| Day | Time | Week -1  1-6.1 | Week 0  7-13.1 | 1th Week  14-20.1 | 2th Week  21-27.1 | 3th Week  28.1 – 3.2 | 4th Week  4-10.2 | 5th Week  11-17.2 | 6th Week  18-24.2 | 7th Week  25.2 – 3.3. | 8th Week  4.3-10.3 | 9th Week  11.3 – 17.3 | 10th week  18.3 – 24.3 | 11 week  25-31.3 |
| --- | --- | --- | --- | --- | --- | --- | --- | --- | --- | --- | --- | --- | --- | --- |
| MON | AM |  | Tec | Tec | Tec | SP |  |  | SP |  |  | Tec | SP | Tec |
|  | PM |  | Tec | Tec | Tec | Tec | Tec | Rec / Tec | Tec |  | M | Tec | Tec | Tac |
| TUE | AM |  | Endurance  Test | Tec / Tac / CG | RE / Tec / / Tac / CG | RE / Tec / / Tac / CG | RE / Tec / / Tac / CG | RE / Tec / / Tac | RE / Tec / / Tac / CG |  | Rec | RE/Tec / Tac / CG | RE/Tec / Tac / CG | Tec / tac / CG |
|  | PM |  |  | MB | MB | MB | MB | MB | MB |  | Tac | MB | MB | MB |
| WED | AM |  | Rec | Rec | Rec | Rec | Rec | Rec | Rec |  | Tac |  | Rec | Rec |
|  | PM |  |  |  |  |  |  |  |  |  |  | Rec |  |  |
| THU | AM | Tec | LB | LB | LB | LB | LB | LB | LB |  | Tec / Tac | LB | LB | LB |
|  | PM | Tec | Tac | RE / Tac | RE / Tac | RE / Tac | RE / Tac | RE / Tac | RE / Tac |  | Tac | RE/Tac | Tac | Tac |
| FRI | AM | LB | Tec |  |  |  | Tec |  |  |  |  |  |  | Tac |
|  | PM | Tec / Tac | Tac | Tac | Tac | Tac | Tac | Tac | Tac | RE / Tec | M | Tac | Tac/CG |  |
| SAT | AM | Familiari-zation and sprint testing | Familiari-zation and sprint testing |  |  |  | OFF |  |  |  | Rec |  |  | Sprint tests |
|  | PM |  |  | M | M | M |  | M | M | Tec / Tac |  | M |  |  |
| SUN | AM |  |  |  |  |  | M |  |  | Tec / Tac |  |  |  |  |
|  | PM |  |  |  |  |  |  |  |  | Tac |  |  |  | M |
| Training hours | | 6h 30min | 9h 40min | 10h | 9h 35min | 9h 30min | 9h 20min | 8h 15min | 9h 25min | 5h 15min | 7h 15min | 9h 50min | 9h 40min | 8h 30 min |
|  |  |  |  |  |  |  |  |  |  |  |  |  |  |  |
