# Supplementary material for: Changes in sprint performance and sagittal plane kinematics after heavy resisted sprint training in professional soccer players
Source: PeerJ. 2020 Dec 15;8:e10507. doi: 10.7717/peerj.10507 (PMC7747683; doi:10.7717/peerj.10507)
Supplement: Supplemental Information 11 — Values: Tec = Technical Training, Tac = Tactical Training, LB = Lower Body, MB = Mixed full body training, CG = Condition Games, Rec = Recovery Training, M = Match [file peerj-08-10507-s011.docx]

| Day | Time | Week 0  7-13.1 | 1th Week  14-20.1 | 2th Week  21-27.1 | 3th Week  28.1 – 3.2 | 4th Week  4-10.2 | 5th Week  11-17.2 | 6th Week  18-24.2 | 7th Week  25.2 – 3.3. | 8 th  4.3-10.3 | 9 th  11.3 – 17.3 | 10 th  18.3 – 24.3 | 11 th  25.3– 31.3 | 12 th week  1-7.4.2019 |
| --- | --- | --- | --- | --- | --- | --- | --- | --- | --- | --- | --- | --- | --- | --- |
| MON | AM | Tec | Tec | Tec | Tec | Tec | Individual  program | Tec | Tec | Tec | Tec | Tec | Tec | Tec |
|  | PM | MB | LB |  |  |  |  |  |  |  |  |  |  |  |
| TUE | AM | Tec (small pitch) | Tec | Team endurance and speed testing | Tac/CG | Tac/Tec | Individual  program | M | Tec/Tac | Tac/CG | Tec/Tac | Tec | Tec/Tac | Tec/Tac |
|  | PM |  | MB |  | LB | LB |  |  | MB | MB |  |  | MB | MB |
| WED | AM | Tec | Tec | Tac | Tac | Tac/CG | Individual  program | MB | Tac/CG | Tec/Tac | Tac/CG | Team endurance and speed testing | Tac/CG | Tac/CG |
|  | PM |  |  |  |  |  |  |  |  |  |  |  |  |  |
| THU | AM | MB | MB | MB | MB | MB | Tec/Tac | Tec | MB | MB | MB | Individual  program | MB | MB |
|  | PM |  |  |  |  |  |  |  |  |  |  |  |  |  |
| FRI | AM | Sprint tests | M | Tac | M | Tac | Tac | Tec | Tec | M | Tec | Individual  program | Tec | Sprint tests |
|  | PM | Tac/CG |  |  |  |  | LB |  |  |  |  |  |  | Tec |
| SAT | AM | MB | Rec | M | Rec MB | M | MB | M | M | Rec | M | Individual  program | M | Tec |
|  | PM |  |  |  |  |  |  |  |  |  |  |  |  |  |
| SUN | AM | Rec | Rec | Rec | Rec | Rec | Rec | Rec | Rec | Rec | Rec | Individual  program | Rec | M |
|  | PM |  |  |  |  |  |  |  |  |  |  |  |  |  |
| Training hours | | 8 h 20 min | 7 h 20 min | 7 h 30 min | 7 h 45 min | 8 h 45 min | 7 h 50 min | 5 h 10 min | 8 h 30 min | 8 h 20 min | 8 h 30 min | 7 h 45 min | 8 h 30 min  9 | 8 h 30 min |
